# Isobavachalcone inhibits Pseudorabies virus by impairing virus-induced cell-to-cell fusion

**DOI:** 10.1186/s12985-020-01312-2

**Published:** 2020-03-17

**Authors:** Yu Wang, Tian-Xin Liu, Tong-Yun Wang, Yan-Dong Tang, Ping Wei

**Affiliations:** 1 College of Veterinary Medicine, Northeast Agricultural University, Harbin, 150001 China; 2 State Key Laboratory of Veterinary Biotechnology, Harbin Veterinary Research Institute of Chinese Academy of Agricultural Sciences, Harbin, 150001 China

**Keywords:** PRV, Isobavachalcone, Antiviral, Fusion

## Abstract

Pseudorabies virus (PRV) is an important pathogen that threatens the global swine industry. Currently, there is no effective drug that can clinically prevent or treat PRV infections. Isobavachalcone (IBC), a natural chalcone compound derived from *Psoralea corylifolia*, displays multiple biological activities, such as antibacterial, antifungal, and anticancer activities. Recently, it was found that IBC exhibited antiviral activity against an RNA virus, porcine reproductive and respiratory syndrome virus (PRRSV), in vitro. In the current study, we further demonstrated for the first time that IBC has a strong inhibitory effect on PRV. Through a viral luciferase expression assay, we showed that the inhibition step occurs mainly in the late stage of viral replication. Finally, via a cell-to-cell fusion assay, we demonstrated that IBC inhibits PRV by blocking virus-mediated cell fusion. Thus, IBC may be a candidate for further therapeutic evaluation against PRV infection in vivo.

## Main text

**Fig. 1:**
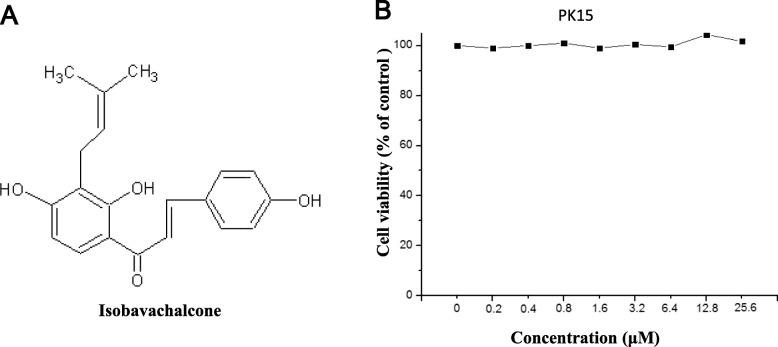
IBC structure and cytotoxicity evaluation. **a** Chemical structure of IBC. **b** Cytotoxicity of IBC in PK15 cells incubated with different concentrations of IBC for 24 h. The cell viability rate was assessed by measuring the absorbance at 490 nm

**Fig. 2:**
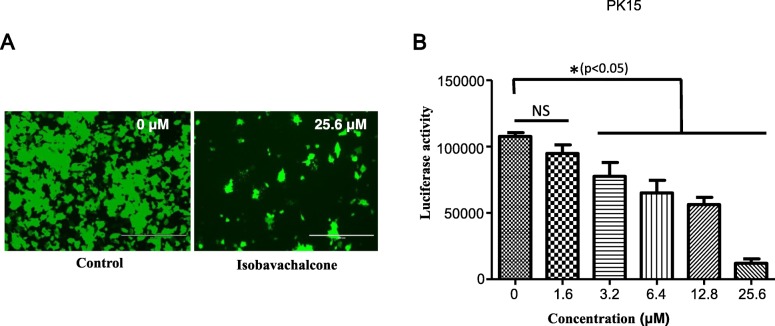
PRV replication is inhibited by IBC. PK15 cells were infected with EGFP- and luciferase-expressing PRV (MOI = 0.1). At 24 h p.i., PRV infection was analyzed by fluorescence microscopy (**a**), and luciferase activity was evaluated (**b**)

**Fig. 3:**
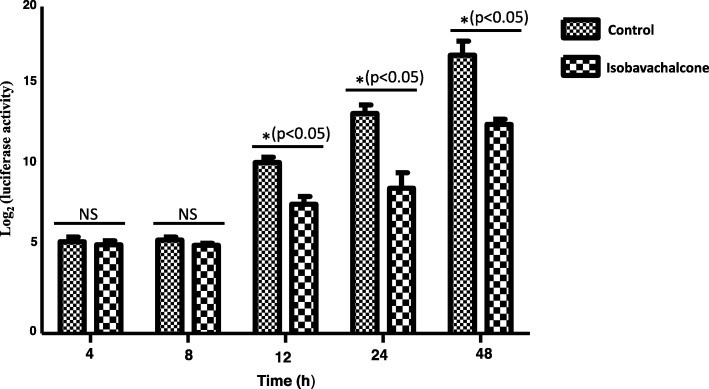
IBC blocks the late stages of the PRV life cycle. PK15 cells were infected with PRV (MOI = 0.1) and incubated in medium containing either ethanol or the indicated doses of IBC. Luciferase activity was evaluated at the indicated time points p.i

**Fig. 4:**
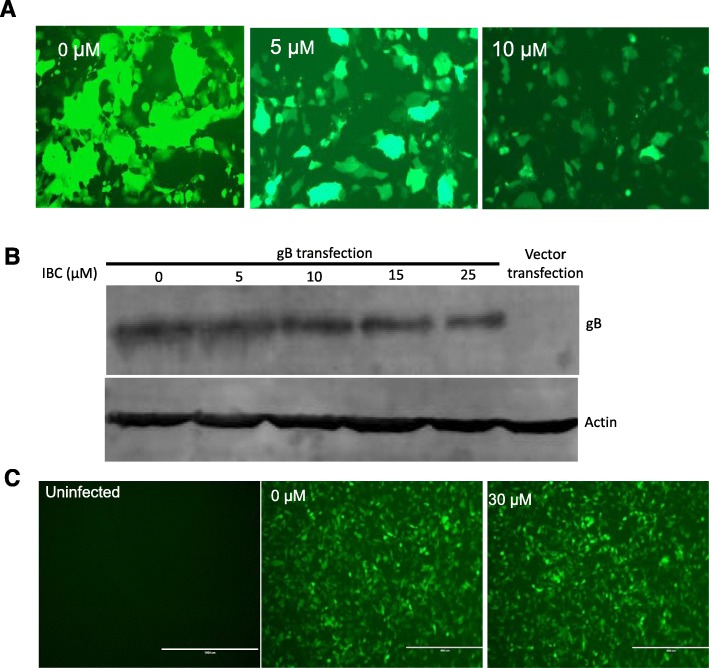
IBC inhibits PRV replication at the cell-to-cell fusion step. **a** RK13 cells were transfected with gB, gH, gL and EGFP expression plasmids, and 6 h later, the medium was replaced with medium containing either ethanol or the indicated doses of IBC. Cell-to-cell fusion was analyzed by fluorescence microscopy. **b** gB expression was evaluated at the indicated IBC concentrations. **c** Adenovirus replication was not influenced by IBC

Pseudorabies virus (PRV) is a swine alphaherpesvirus that causes Aujeszky’s disease in pigs [1, 2]. PRV poses a serious threat to the pig industry, especially since novel PRV variants began emerging in 2011 [3, 4]. Most importantly, recent studies have reported that humans can be infected by PRV [5–7], indicating that PRV is also a potential threat to humans [6]. Thus, exploring new anti-PRV agents may be an effective means of controlling PRV. In this study, we explored the potential anti-PRV activity of isobavachalcone (IBC), a traditional Chinese medicine (TCM). The structure of IBC is shown in Fig. 1a. IBC was first isolated from *Psoralea corylifolia* in 1968 and possesses a broad spectrum of biological activities [8]. In a recent study, IBC also exhibited anti-porcine reproductive and respiratory syndrome virus (PRRSV) activity during the early stage of viral RNA synthesis [9]. To explore whether IBC has anti-PRV activity, we first evaluated the cytotoxicity of IBC on PK15 cells with a Cell Counting Kit-8 (CCK8, Dojindo Laboratories, Japan) according to the manufacturer’s instructions. Cell viability was not changed relative to that of control cells at IBC concentrations of up to 25 μM. Next, we evaluated the antiviral activity of IBC via a recombinant PRV reporter virus expressing both enhanced green fluorescent protein (EGFP) and firefly luciferase [10]. These results showed that both EGFP expression and luciferase activity were significantly decreased in the IBC-treated group compared to the control group (Fig. 2a and b), indicating that PRV replication was significantly inhibited. However, the step in PRV replication that is influenced by IBC is unknown. To determine which step(s) in the viral life cycle are affected, we first treated PK15 cells with IBC and infected them 2 h later with the PRV reporter virus at an MOI of 0.01. IBC markedly inhibited PRV from 12 to 48 h post infection (p.i.), whereas PRV replication was not influenced at 4 and 8 h p.i. (Fig. 3). According to the results of the one-step growth curves for the pseudorabies viruses, the virus titer reached a peak approximately 14 h post infection, so we recognized that 4 and 8 h post infection was the early stage. This pattern indicated that IBC inhibits PRV during the late stage of the viral lifecycle. In addition, these results demonstrated that the mechanisms by which IBC inhibits PRV and PRRSV may differ because IBC inhibited PRRSV at an early stage in our previous study [9]. PRV can induce cell-to-cell fusion, a very important step in viral spreading, at a late stage in the lifecycle. Here, to investigate whether IBC inhibits PRV replication at this stage, we performed transient transfection-based cell-to-cell fusion assays, as previously described [11, 12]. Briefly, RK13 cells were transfected with 200 ng each of the EGFP expression plasmid (pDC315-EGFP [13]) or expression plasmids for the gB, gL, and gH PRV glycoproteins (constructed in this study by subcloning these genes into the pCAGGS-HA vector) with Lipofectamine 2000 according to the manufacturer’s instructions. Six hours later, the cell culture medium was replaced with medium with or without IBC, the cells were fixed 24 h post transfection, and syncytium formation was analyzed by fluorescence microscopy. Consistent with our hypothesis, IBC significantly inhibited PRV glycoprotein-induced cell-to-cell fusion (Fig. 4a). To exclude the possibility that the cell-to-cell fusion was caused by inhibition of gB expression by IBC, we next evaluated gB expression with a gB-specific monoclonal antibody (1:200; the gB mAb was kindly provided by Professor Zhi-Jun Tian of the Harbin Veterinary Research Institute of the Chinese Academy of Agricultural Sciences). We found that after IBC treatment, gB expression was not influenced by IBC (Fig. 4b). The above results showed that IBC inhibits PRV replication mainly at the cell-to-cell fusion step in the late stage of the lifecycle. To further confirm this finding, we evaluated IBC activity against nonenveloped adenoviruses. If IBC inhibits adenovirus replication, it may also play an antiviral role in steps of the lifecycle other than cell-to-cell fusion. An EGFP-expressing adenovirus [13] was used to infect HEK293 cells (MOI = 0.1) in this study. Twenty-four hours later, the results indicated that adenovirus was not inhibited by IBC in HEK293 cells (Fig. 4c), further confirming that IBC inhibits PRV replication at the cell-to-cell fusion step. In our previous study, we demonstrated that the CRISPR/Cas9 system may be a powerful tool for PRV inhibition and elimination [14, 15]. However, this method requires gene transfer tools to introduce components of the CRISPR system into the cell. Thus, antiviral agents such as IBC may be more suitable for virus control in the current setting.

In conclusion, we showed that IBC exhibits antiviral activity against PRV and demonstrated that IBC treatment significantly blocked PRV-mediated cell-to-cell fusion. Therefore, IBC may be a candidate for further therapeutic evaluation against PRV infection in swine.

## Data Availability

Not applicable.

## Abbreviations

PRV: Pseudorabies virus
GFP: Green fluorescent protein
IBC: Isobavachalcone
TCM: Traditional Chinese medicine
PRRSV: Porcine reproductive and respiratory syndrome virus
